# Introduction to Editorial Board Member: Professor W. Mark Saltzman

**DOI:** 10.1002/btm2.10174

**Published:** 2020-08-05

**Authors:** Steven M. Jay

**Affiliations:** ^1^ Fischell Department of Bioengineering and Program in Molecular and Cell Biology University of Maryland College Park Maryland USA

In this issue of *Bioengineering and Translational Medicine*, we are pleased to introduce our Editorial Board Member, Professor W. Mark Saltzman. Professor Saltzman is the Goizueta Foundation Professor of Biomedical Engineering, Chemical and Environmental Engineering, and Physiology at Yale University. Professor Saltzman received his BS in chemical engineering from Iowa State University before earning a SM in chemical engineering and a PhD in medical engineering from the Massachusetts Institute of Technology (MIT).

**FIGURE 1:**
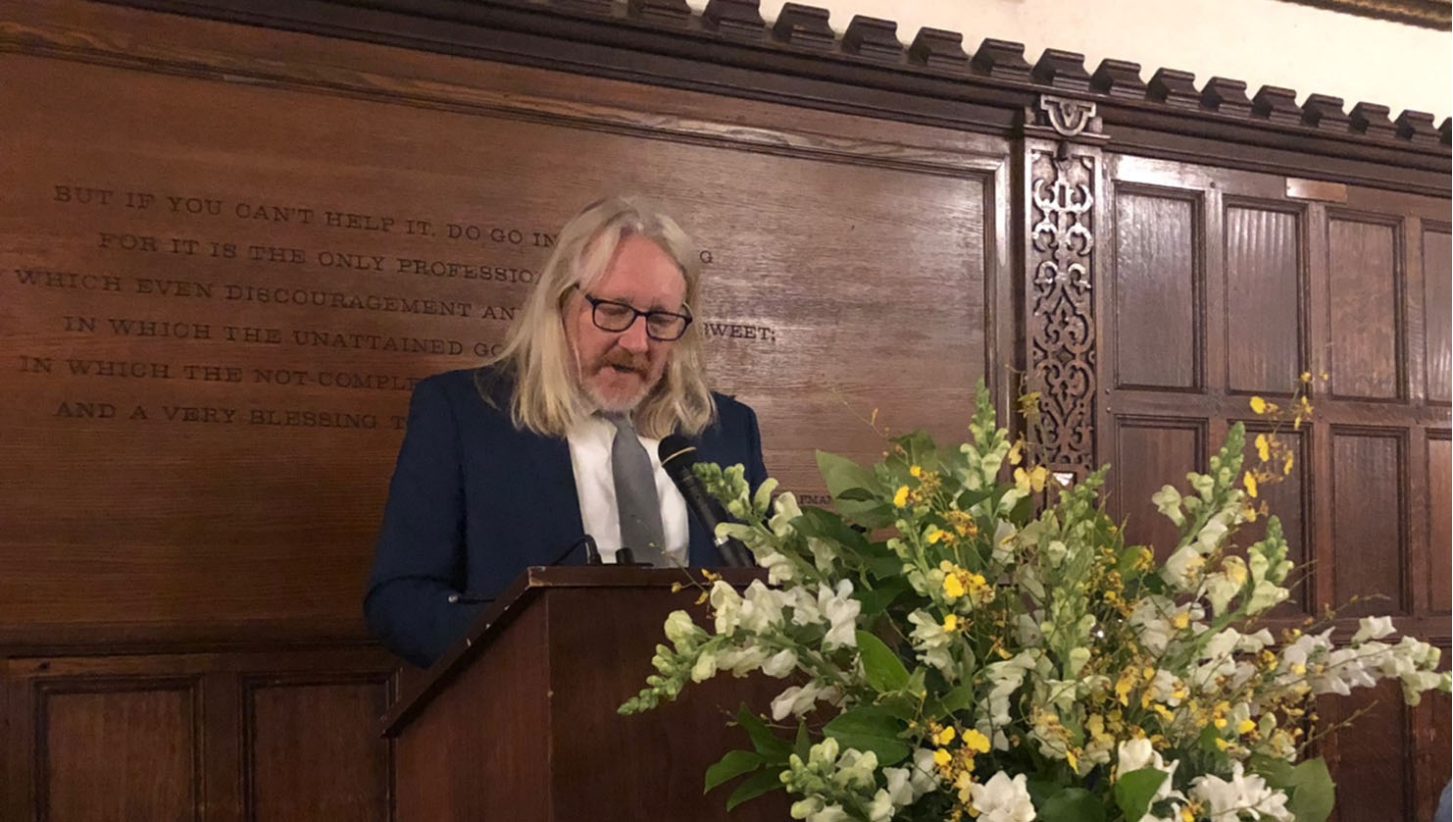
Professor Saltzman addresses the audience at a dinner celebration in 2019 as part of a research symposium on the occasion of his birthday. Photo credit: Jennifer Saucier‐Sawyer

Professor Saltzman's research has been formative in the fields of controlled drug delivery and tissue engineering. As a trainee in the laboratory of Professor Robert Langer at MIT, Professor Saltzman participated in some of the very earliest work in utilizing polymer scaffolds for cell transplantation with Dr Joseph Vacanti.^1^ Following the establishment of his independent lab, first at Johns Hopkins University and later at Cornell and Yale Universities, Professor Saltzman extended the knowledge of the field on the interaction of cells with polymer and hydrogel matrices. In particular, his work applied engineering principles to understand cell mobility and migration within implantable matrices,^2^, ^3^, ^4^ establishing a basis for quantitative design of engineered tissues. Another major focus has been the integration of drug delivery into tissue engineering and cell transplantation approaches.^5^ Long‐standing collaborations with Professor Jordan Pober and others at the Yale School of Medicine have explored how controlled delivery approaches can enhance vascular tissue repair and regeneration outcomes (Figure 1).^6^, ^7^, ^8^, ^9^, ^10^

Professor Saltzman is recognized as a pioneer in quantifying and modeling the transport of macromolecules through synthetic polymers and biological matrices,^11^, ^12^ having applied this work in particular to female reproductive health applications^13^, ^14^ and to the treatment of neurodegenerative diseases and brain cancer.^15^, ^16^ His early career work in this area was recognized with the Allan C. Davis Medal as Maryland's Outstanding Young Engineer (1995) and the Controlled Release Society's Young Investigator Award (1996). Professor Saltzman's research group has proceeded to make numerous fundamental discoveries about how drug carriers interact with cells,^17^, ^18^, ^19^ which have informed the development of innovative delivery systems for small molecules, proteins, and nucleic acids.^20^, ^21^, ^22^, ^23^ Several of these efforts have moved beyond the lab towards clinical translation, and Professor Saltzman was recognized as a Fellow of the National Academy of Inventors in 2013.

**FIGURE 2:**
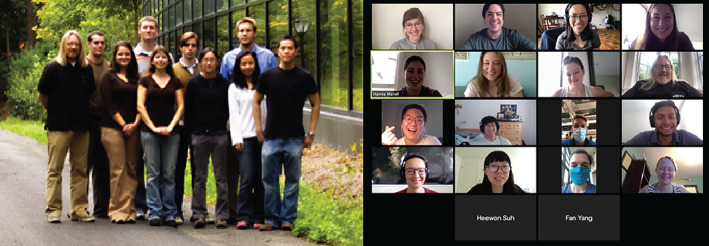
Professor Saltzman's research group in ∼2007 (left) and 2020 (right). Photo credit: Mark Saltzman

In addition to his research excellence, Professor Saltzman is passionate about education and has left an indelible mark in this area as well. He has contributed to the emergence of the academic field of biomedical engineering by authoring three original textbooks^24^, ^25^, ^26^ and an online course, all of which have been used by numerous instructors and students at institutions across the world. Professor Saltzman has also served as Head of Jonathan Edwards College, one of 14 residential colleges at Yale, where he lives in residence with students and serves as a mentor in all areas of student life. Professor Saltzman's education contributions have been recognized by the Camille and Henry Dreyfus Foundation Teacher‐Scholar Award (1990) and Yale's Sheffield Teaching Prize for excellence in the classroom (2009) (Figure 2).

Over the past 30+ years, Professor Saltzman's contributions as a researcher, educator, and mentor have been immense. He has supervised the research work of over 43 PhD students and 27 postdoctoral researchers who now populate the academic and industry landscape and contribute to his legacy of fundamental discovery and translation in bioengineering. His overall excellence has been recognized via election as Fellow of the American Institute for Medical and Biological Engineering (1997), Fellow of the Biomedical Engineering Society (2010), member of the U.S. National Academy of Medicine (2014), and member of the U.S. National Academy of Engineering (2018). Moreover, Professor Saltzman has set a standard for supportive mentorship that has created a family‐like network among his trainees. He is always available for advice and support, even for those who have long ago left his group, no matter how many other demands on his time there may be. On behalf of his current and former trainees, I express my appreciation and gratitude to Mark for sharing his scientific journey with us and helping to shape our own through his insight and guidance.
